# Mobile Phones and HIV Testing: Multicountry Instrumental Variable Analysis From Sub-Saharan Africa

**DOI:** 10.2196/48794

**Published:** 2024-09-27

**Authors:** Francesco Iacoella, Nyasha Tirivayi

**Affiliations:** 1 UNICEF Evaluation Office New York, NY United States; 2 UNICEF Innocenti-Global Office of Research and Foresight Florence Italy

**Keywords:** information and knowledge, communication, health and economic development, public health, technological change, choices and consequences, mobile phone, connectivity, access, HIV, testing, Sub-Saharan Africa, women’s health

## Abstract

**Background:**

Sub-Saharan Africa has been a technological hothouse when it comes to mobile phone technology adoption. However, evidence on the role played by mobile technology on infectious disease prevention has been mostly limited to experimental studies.

**Objective:**

This observational study investigates the role of mobile phone connectivity on HIV testing in sub-Saharan Africa.

**Methods:**

We make use of the novel and comprehensive OpenCelliD cell tower database and Demographic and Health Survey geocoded information for over 400,000 women in 29 sub-Saharan African countries. We examine, through ordinary least square and instrumental variable regressions, whether women’s community distance from the closest cell tower influences knowledge about HIV testing facilities and the likelihood of ever being tested for HIV.

**Results:**

After finding a negative and significant impact of distance to the nearest cell tower on knowledge of HIV testing facility (–0.7 percentage points per unit increase in distance) and HIV testing (–0.5 percentage points per unit increase), we investigate the mechanisms through which such effects might occur. Our analysis shows that distance to a cell tower reduces HIV-related knowledge (–0.4 percentage points per unit increase) as well as reproductive health knowledge (–0.4 percentage points per unit increase). Similar results are observed when the analysis is performed at community level.

**Conclusions:**

Results suggest that the effect of mobile phone connectivity is channeled through increased knowledge of HIV, sexually transmittable infections, and modern contraceptive methods. Further analysis shows that cell phone ownership has an even larger impact on HIV testing and knowledge. This paper adds to the recent literature on the impact of mobile-based HIV prevention schemes by showing through large-scale analysis that better mobile network access is a powerful tool to spread reproductive health knowledge and increase HIV awareness.

## Introduction

Sub-Saharan Africa carries a disproportionate burden of HIV infections and AIDS-related diseases. In 2016, of the 6000 new HIV infections happening globally every day, 2 out of 3 occurred in sub-Saharan Africa [1]. Global AIDS-related deaths have sharply decreased in the past decade, with sub-Saharan African countries leading this downward trend [2], largely due to the increased access to antiretroviral drugs. AIDS-related mortality has decreased by approximately 40% since 2010 and by 60% since it reached its peak in 2004 [2]. However, the number of new infections has not decreased at the same pace. Although the number of new HIV infections decreased by approximately 23% worldwide since 2010, with Southern and Eastern Africa showing the largest reduction, in 2019, there were over 1.7 million new infections, of which 1.4 million happened in sub-Saharan Africa (and 59% of whom are women) [2]. More importantly, not every person living with HIV is aware of her condition [3]. Estimates for people aware of their condition in sub-Saharan African countries range between 45% [4] and 65% [3]; hence, the numbers of infected individuals are potentially being underestimated by the hundreds of thousands. To increase the capacity of governments to correctly identify HIV-infected individuals and therefore reduce the spreading of the virus, standard HIV-testing facilities and awareness-raising campaigns are being paired with alternative methods such as community-based testing, self-test kits, and point-of-care testing for early infants [3]. Major barriers to testing (eg, stigma, inaccessibility of services, and low awareness) are being gradually overcome by these new techniques, which are currently being rolled out in numerous sub-Saharan countries [3-5]. Expanding HIV-related knowledge and introducing optimized HIV-testing techniques such as community-based testing and self-testing can potentially increase the returns from national anti-HIV programs [6].

Sub-Saharan Africa has proven to be a technological hothouse when it comes to the expansion of mobile phone coverage and use. The rapid increase in mobile subscribers’ numbers and the expansion of network penetration have resulted in sub-Saharan Africa becoming the world’s fastest-growing mobile region. Mobile phones facilitate individuals’ access to information from sources that would otherwise be unreachable or costly and enable near-instant communication through voice and text (or video, in the case of smartphones). Mobile phone penetration had a pivotal role in the region, influencing economic growth and human development [7,8]. It has been argued that the “communication” component of mobile phones holds the most potential of supporting economic development by creating knowledge-sharing networks [9-11]. Recently, researchers have been investigating the role of mobile phones in public health and found them to be powerful awareness-raising tools and facilitators of individuals’ communication with health workers and other patients [12-14]. For mobile phone users, new information is, therefore, acquired mostly through communication with other individuals. However, mobile phones can also facilitate access to institutional resources (eg, by receiving informative SMS text messages from a health clinic, listening to radio, or, for those with access to a smartphone, surfing the internet). Evidence from rural Indonesia has shown that midwives’ use of mobile phones increased access to institutional resources and, consequently, fostered better reproductive health knowledge. Further, access to peer resources was associated with higher self-efficacy, which was as well positively associated with health knowledge [15]. Mobile phone ownership has been found to significantly influence contraception use and reproductive health attitudes [16,17]. Phones have also been used in recent years as a new way of reaching individuals with or at risk of HIV with awareness-raising campaigns (see Project Masiluleke in South Africa and Text to Change in Uganda). Finally, recent evidence identified mobile phone ownership as a more relevant determinant of contraceptive use and reproductive health care access than television or radio [18].

Some studies have focused on investigating mobile-based, awareness-raising messaging and adherence to HIV/AIDS treatment programs through randomized controlled trials [19-23]. Lester et al [20], in a seminal study on this topic, examined the association between higher adherence to antiretroviral treatment and SMS text messaging–based support in Kenya. More recent studies have questioned the role of SMS text messaging–based support as a “silver bullet” in resource-limited settings, especially after second-line antiretroviral treatment failure, although it still remains a valuable tool for promoting higher participation in anti-HIV/AIDS programs in general [19]. A study on the role of mobile apps for patients newly diagnosed as HIV-positive found that young individuals in particular are more responsive to mobile-based treatment adherence campaigns [20], while another is planning to test an innovative self-testing service that uses artificial intelligence [23]. However, the impact of mobile technology on HIV and HIV-testing knowledge and uptake might go way beyond the effectiveness of SMS text messaging–based or app-based support.

The lack of literature on the topic makes it difficult to formulate hypotheses on the role played by cellular phones, although it has been shown that innovative, mobile-based, awareness-raising campaigns at community and individual levels represent an economically sensible alternative to traditional awareness-raising campaigns [3]. Moreover, mobile technology–enabled networks facilitate peer-to-peer communication, which has been proven to increase HIV knowledge and treatment adherence while reducing HIV risk [24-26]. Finally, mobile devices may accord privacy in communication, which could facilitate the discussion of a topic such as HIV prevention, which is still stigmatized. We hypothesize that mobile-enabled communication increases HIV- and reproductive health–related knowledge and, through that, it affects HIV-testing knowledge and uptake.

Considering the high number of new infections that happen every year in sub-Saharan African countries and the fact that a large portion of people living with HIV are still unaware of their condition, it is crucial to better understand if and how mobile technology can support HIV prevention and testing. Although there is an emerging body of evidence linking mobile phones with health promotion and awareness, to our knowledge, evidence on the role played by mobile phone ownership or connectivity on HIV-testing knowledge and attitudes, and an investigation of impact pathways, is lacking. Our study seeks to address this knowledge gap. It examines the role mobile connectivity can play in promoting HIV testing through multicountry analysis in sub-Sahara Africa. We focus on mobile connectivity instead of mobile ownership to account for the fact that phone sharing is very common in sub-Saharan African countries [9,13]. Our study is, to our knowledge, the first of its kind to establish a connection between widespread access to mobile phone network and knowledge of HIV-testing facilities and ultimately HIV-testing uptake. We use recent data on cell tower locations and data on women’s HIV testing and knowledge in 29 sub-Saharan African countries. In our paper, we also explore the mechanisms through which mobile phone connectivity affects HIV-testing knowledge and uptake. We test if mobile connectivity has a direct effect on levels of HIV knowledge and whether it influences overall knowledge about reproductive health (ie, knowledge about other sexually transmittable infections [STIs] and about contraceptive methods). We find that indeed mobile connectivity influences general knowledge about HIV and reproductive health.

With our research, we establish a causal link between mobile network connectivity and HIV-testing knowledge and practices using individual-level data from 29 African countries, and we shed light on potential mechanisms for these results. Our work fills a significant gap in HIV-testing literature by providing observational evidence and investigating the role of both mobile connectivity and mobile phone ownership.

## Methods

### Data

Several data sources have been used to build the dataset used in our analysis.

#### Cell Connectivity Information

**Figure 1:**
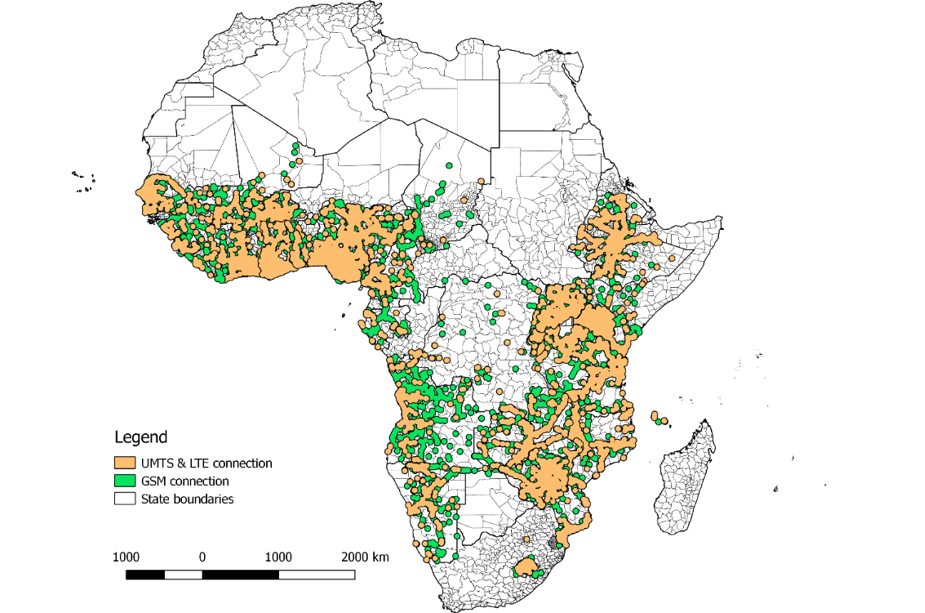
Cell phone coverage in sub-Saharan African countries included in our study. Coverage is calculated as a buffer of 35 km around each cell tower for the 29 countries included in the dataset. The image is the authors’ elaboration of OpenCelliD data. GSM: Global System for Mobile Communication; LTE: long-term evolution; UMTS: Universal Mobile Telecommunications System.

Information on the location of cell towers is derived from the OpenCelliD dataset. OpenCelliD is the largest, daily updated, publicly available database of cell towers. The linear distance between household clusters and the nearest cell tower is calculated in kilometers and serves as a proxy for cell connectivity and signal strength and as our main explanatory variable. OpenCelliD dataset reports the location, operator, and type of signal for over 40 million towers worldwide. Database entries for the African continent (Figure 1) started in 2008. Unfortunately, the date of entry does not unequivocally represent the date of construction of a tower. Because of this, many countries show a total absence of cell towers before 2013, a clearly misleading representation. This is why we calculate the distance from the nearest cell tower including all cell towers in the country regardless of the date in which they have been added to the OpenCelliD dataset. This is done because we assume that towers have most probably been built before their date of entry in the dataset. Since this is a very strong assumption, we test its robustness by running our analysis again, this time limiting the number of cell towers per country to those added to OpenCelliD before the year in which individual survey data were collected. As we will show in the *Results* section, results remain consistent even when the number of cell towers (and, therefore, of countries) is reduced. One more issue with OpenCelliD data is that the tower location is not verified after it is inserted in the database, which might result in a discrepancy between reported and actual location. However, one study comparing independently collected data on cell tower coordinates and OpenCelliD information on the same towers shows that the localization error estimated from OpenCelliD data agrees well with the experimental error distribution [27]. As an extra precaution, however, we consider only towers whose coordinates’ maximum error (reported by OpenCelliD) lies within 1 km^2^.

#### Individual- and Community-Level Data

Individual data are obtained from the Demographic and Health Survey (DHS) program from 29 African countries—Angola, Benin, Burkina Faso, Burundi, Cameroon, Chad, Comoros, Ivory Coast, Democratic Republic of Congo, Ethiopia, Gabon, Ghana, Guinea, Kenya, Lesotho, Liberia, Malawi, Mali, Mozambique, Namibia, Nigeria, Rwanda, Senegal, Sierra Leone, Tanzania, Togo, Uganda, Zambia, and Zimbabwe. DHS provides cross-sectional information on women and household access to health and health status together with demographic and labor statistics. Besides women’s demographic and employment information, we take from DHS our main dependent variables: a dummy for knowledge of facilities where women could be tested for HIV and a dummy for ever been tested for HIV. We use additional variables on knowledge of reproductive health to test our hypothesis on possible impact pathways. Individual-level characteristics are added to the analysis to serve as confounders. We include information on the women’s age, education, employment, marital status, if ever had children, and exposure to media (ie, newspapers, radio, or television). We also include household-level indicators of gender of the household head, place of living (ie, urban or rural), a wealth index built by DHS and based on selected assets, dwelling construction materials, and types of water access and sanitation facilities. Finally, to address the role played by access to health facilities in facilitating HIV testing, we include in our analysis 2 dummy variables: the first reporting value one for women having visited a health facility in the past 12 months and the second reporting value one for perceiving distance to health facilities as a problem. However, evidence shows that perception of distance might play a bigger role than actual distance when deciding whether to go to a health facility for testing [28]). For each country, DHS included in this study has been conducted between 2010 and 2016. The final sample comprises 407,000 women grouped in 19,603 clusters, of whom, approximately 350,000 are included in the main analysis. As DHS is conducted to be representative at the country level, our study provides estimates relevant for the entire population of the countries involved in it.

Geospatial information of households in DHS datasets is clustered to guarantee respondents’ privacy. For these clusters, DHS provides spatial information retrieved from secondary sources within a buffer of 10 km for rural communities and 2 km for urban ones. We include in the analysis community-level (ie, cluster-level) information on average travel time to the nearest settlement (ie, an urban area with at least 50,000 inhabitants) and population density. We also use household-level information on access to electricity to generate a community-level electrification rate indicator. This indicator is simply the percentage of households who have access to electricity within a cluster.

Living a long distance from the nearest cell tower might be one of many indicators of a community’s geographical isolation. More isolated communities could have lower level of HIV testing regardless of their use of mobile phones [29]. To better measure community isolation and to control for it in the analysis, we calculate the linear distance of each household’s cluster from the nearest primary or secondary road (trails and nonpaved routes have not been considered in the analysis). Information on roads in the African continent is obtained through the Digital Chart of the World (DCW) vector basemap. Although last updated in 1998, DCW remains a very detailed map of Africa’s road system. Authors have considered using more recent street maps for the analysis; however, they ultimately decided to make use of DCW due to its reliability. National Aeronautics and Space Administration Global Roads Open Access Dataset, although more recent, does not provide users the chance to adequately distinguish types of roads in the African continent. However, for those countries in which a comparison between DCW and Global Roads Open Access Dataset was possible (eg, Zimbabwe), the authors verified that primary roads reported in the latter were also present in the former (graphical representation in Figure S1 in Multimedia Appendix 1). Distance to the road is used, together with distance to the nearest settlement and community-level electrification rate, as a proxy for community isolation.

Finally, we include information on land gradient and HIV prevalence for each of the clusters. This information is obtained from DHS elaboration of the United States Geological Survey GTOPO30. HIV prevalence (as a number of people who are HIV positive over 100,000) within a 10-km radius from the cluster is obtained from the mapping exercise by Dwyer-Lindgren et al [30], which produced a 5×5-km grid of HIV prevalence among adults for sub-Saharan Africa. Grids are provided for every year between 2000 and 2017. Since 2010 represents the less recent years in which surveys included in our dataset were collected, we use HIV prevalence information from that year. Data are analyzed using QGIS (QGIS Development Team) and Stata (version 16; StataCorp LLC) software.

#### Sample Characteristics

Table 1 reports descriptive statistics from the sample. The average community distance from the cell tower is 13.06 km. This is considering both rural and urban areas. The average woman’s age is 28.46 years, with 5 years of education. About 28.34% of interviewed women live in a female-headed household, 72.05% are exposed to at least 1 type of media (ie, radio, television, and newspaper), 52.34% are in a union, 58.60% are currently working, and 35.40% live in an urban area. Half of the women in the sample report to have visited a health facility in the last 12 months, although distance from said facility is considered a problem by 41.04% of them. A total of 72.66% of sampled women have given birth to at least 1 child. The average population density in the communities was 1290 people per km^2^ (this is considering a buffer of 2 km^2^ for urban areas and 10 km^2^ for rural areas, 2015 population data). Distance from the closest road (trails and nonpaved routes have been excluded from the analysis) is 6.12 km on average, and the average travel time to the closest settlement is about 77.48 minutes. Finally, the prevalence of households with access to electricity in the community is about 31.81% on average.

**Table 1:** Descriptive statistics of the sample.

Variables		Values		Total, N	
Distance from the nearest cell tower (km), mean		13.06		517,290	
Woman age, mean		28.46		517,290	
Woman years of education, mean		5.18		517,110	
Female-headed households (%)		28.34		517,290	
Woman exposed to any type of media (%)		72.05		517,257	
Woman currently working (%)		58.60		500,001	
Ever had a child (%)		72.66		517,290	
Number of lifetime sex partners, mean		2.16		407,542	
Woman in union (%)		52.34		517,284	
Living in urban area (%)		35.40		517,290	
Population density in community (2015)^a^, mean		1290.64		516,411	
**Wealth index quintiles** **(%)**					517,290
	Poorest	19.42			
	Poorer	18.56			
	Middle	19.01			
	Richer	19.96			
	Richest	23.05			
Travel time to closest settlement (minutes)^a^, mean		77.48		516,411	
Distance from the closest road (km)^a^, mean		6.12		517,290	
HIV prevalence in 10-km radius, mean		5.05		517,022	
Visited a health facility in the past 12 months (%)		50.31		499,767	
Distance to a health facility is considered a problem (%)		41.04		458,976	
Electrification (cluster level)^a^ (%)		31.81		517,290	

^a^Community-level variables obtained by averaging household figures at the community level.

### Statistical Methods

Using probit and ordinary least square regressions, we design a naive model at first.

HIV*t_i_*=β_0_+β_1_*D_i_*+β_2_*Z_i_*+β_3_*C*+*u_i_*
**(1)**

Where HIV*t_i_* is a dummy variable indicating if either woman knows where the HIV-testing facility is or has ever been tested for HIV. β_1_ is a vector of our main explanatory variable or distance to nearest cell tower *D* for woman *i*. β_2_ is a vector of covariates *Z* for woman *i*. A full list of covariates is present in Table 1. Finally, β_3_ is a vector of community-level characteristics and country-fixed effects *C*. Location information, from which distance from cell towers was calculated, could only be obtained at the community level. Community clusters contain between 1 and 92 households each. To address intracluster correlation, we cluster SEs at the community level in each regression. Common practice is to cluster SEs at the most aggregate level [31]. However, it is important to consider that in our case sampling has been done at the cluster level and, more importantly, the treatment (ie, distance from the cell tower) is measured at the community level. Correlation in SEs at the community level is, therefore, very plausible, and clustering errors at a higher level might be unnecessarily conservative [32]. The authors decided to present results with SEs clustered at the community level to address such correlation. However, robustness checks have been performed by clustering errors at the country and administrative area levels. Both models produced similar and significant results (results available upon request). Denormalized individual sample weights are included. Individual women’s weights have been calculated by denormalizing DHS individual weights following Ruilin Ren’s note on DHS weight denormalization. As a robustness check, analysis has been run without weights, and results remain consistent (results available upon request).

**Figure 2:**
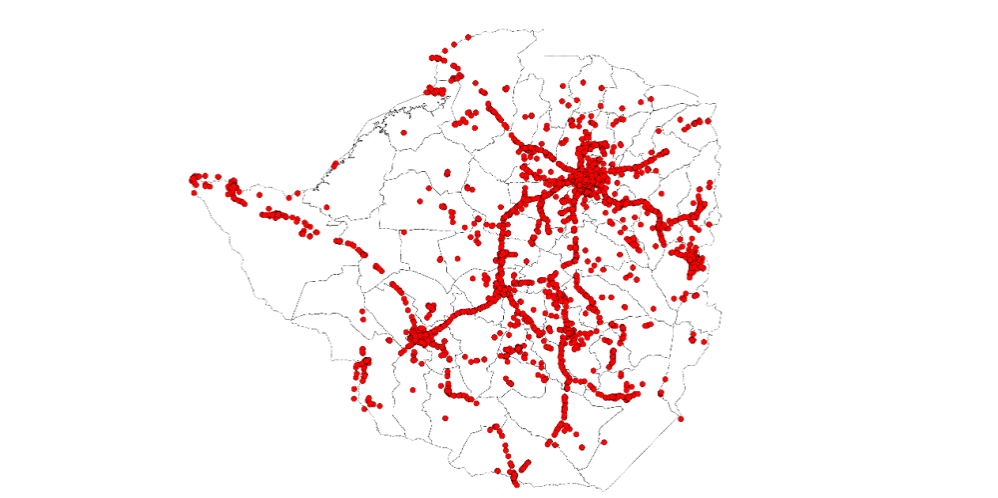
Distribution of cell towers in Zimbabwe. The image is the authors’ elaboration on OpenCelliD data.

As Klonner and Nolen [33] observe, the distribution of cell towers is not exogenous over a country’s territory. It might follow an established path (ie, roads) and be affected by the power supply and other infrastructural needs. Moreover, network providers might consider certain areas not profitable enough to repay them for the investment of building a cell tower. Figure 2 shows this endogenous distribution in the context of Zimbabwe.

To deal with endogeneity, we look for an instrument that could influence towers’ location but is uncorrelated with our dependent variable error terms. Following Dinkelman et al [34], we use the average community land gradient as an instrumental variable (IV). Terrain slope raises the costs of electric line routing and antennas’ building [33,35,36], acting as a disincentive for mobile network companies to build cell towers in areas with higher land gradient. On the contrary, more competitive markets in sub-Saharan Africa can lead to a considerable increase in cell phone area coverage [37]. Proxies for market competition have been used in the past in tandem with terrain roughness to explain the positioning of cell towers [38]. We build our IV interacting land gradient and number of phone service providers in each country following Berman et al [39]. We add to the list of covariates information on household electrification. In this way, we control for effects related to access to electricity regardless of whether a cell phone network is available. Our second model, a 2-stage regression is, therefore:

*D_i_* = π_0_ + π_1_*Slope* × *Network providers_i_* + π_2_*Z_i_* + π_3_*Elec_i_* + π_4_*C* + ε*_i_*
**(2)**

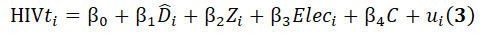



In equation 2, the measure of land gradient *Slope* multiplied by number of *Network providers* is the exogenous regressors. Electrification *Elec* ranges between 0 and 1 and represents the percentage of households who have access to electricity in the community. Equation 3 is similar to equation 1, although here distance *D* has been instrumented by *Slope*×*Network providers*. The land gradient is calculated by DHS based on the United States Geological Survey Global 30 arc-second elevation model (GTOPO30) [40], and we consider it invariant through time (or, at least, within the same geological era). This model is estimated via 2-stage least squares (2SLS).

**Figure 3:**
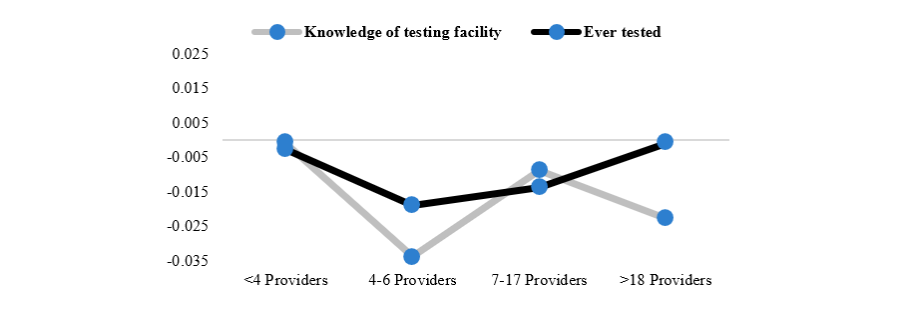
Placebo test. Number of providers correspond to, from left to right, the 50th percentile of the sample, the 75th percentile, the 90th percentile, and the 100th percentile.

The identification assumption is that, conditional on individual and community characteristics, our IV does not directly affect knowledge of facilities or decision to test for HIV and only does so through the distance from the nearest cell tower. To check our assumption, we perform several placebo tests. Mobile phone signal spreads unevenly in the area surrounding cell towers due to a variety of factors. Given the geographical extent of our analysis, we are not able to calculate network coverage exactly. However, technical limitations of standard Global System for Mobile Communication (GSM) towers set their maximum signal range to a 35-km radius; therefore, communities located more than 35 km away from the closest cell tower (9.23% of the sample) can be considered not covered by the mobile network. The 35-km maximum range is the one imposed by technical limitations on GSM towers; however, it does not take into account barriers to signal that might be present within the radius of the cell tower. This is why the dummy build with this cutoff point is used only as an addition to our main explanatory variable (ie, distance from the cell tower). See 3GPP for additional information on timing advance and time division multiple access technology and the way they influence GSM tower range. For the sake of standardization, we assume towers do not possess features to extend their range. Using this cutoff point, which theoretically divides the sample into connected and nonconnected communities, we examine whether our IV only affects outcomes when communities are covered by a mobile network. Results (Table S1 in Multimedia Appendix 1) demonstrate that our instrument is only correlated with outcomes in network-covered communities. Our second test investigates whether the IV or instrumented distance from the cell tower is in any way correlated to proxies of communities’ remoteness, a factor that could in turn affect knowledge of HIV testing place and test uptake. We select 3 different proxies: distance from the nearest road (excluding trails and unpaved roads), exposure to any type of media as proxies for remoteness, and average population density in the community as a proxy for dispersion. Both the instrument and the instrumented distance from the nearest cell tower show no correlation with any of the proxies, supporting our initial assumption that a longer distance from a cell tower is not necessarily related to the remoteness of a community (Table S2 in Multimedia Appendix 1 for full results). Finally, we test the sensibility of our IV. Our results might be driven by countries where markets are more accessible for mobile phone providers. Competition might decrease mobile technology consumption costs and facilitate knowledge sharing. We run our models once again, this time dividing the sample based on the number of mobile network providers in the country of residence. Results remain consistently negative throughout the models (Figure 3), confirming that the relationship between distance from cell towers and our outcomes does not change based on mobile network market characteristics.

### Ethical Considerations

No ethical approval was required for this study as it uses secondary data from DHS. These data are available from DHS on request and received approval from the International Coaching Federation Institutional Review Board. Study data are anonymous.

## Results

### Mobile Phone Connectivity and Knowledge of HIV-Testing Facilities

Our analysis examines whether better mobile connectivity is correlated with women’s knowledge of which facilities offer HIV testing. Results are presented in Table 2. Probit regression results show a strong negative correlation (*P*<.001) between distance from the cell tower and knowledge of an HIV-testing facility. Results from the 2SLS estimation are consistent with our probit results. Due to the nature of the variables, interpreting the magnitude of the impact is not straightforward. We could infer that for each unit increase in distance to the nearest cell tower, the probability of knowing where one can be tested for HIV is 0.7 percentage points lower (0.2 percentage points for probit analysis). To facilitate the interpretation of results and to put them in the right order of magnitude, we report (in Figure S2 in Multimedia Appendix 1) a graphical illustration of our IV model’s predicted share of women knowledgeable about testing facilities by deciles of distance to the nearest cell tower. As it can be noticed, 90% or more of women living in the top deciles are knowledgeable about HIV-testing facility locations. The share starts dropping starting from the fourth decile, going below 80% in the bottom 2 deciles (ie, with a distance >15 km), and reaching 40% in the lowest decile (ie, distance >33 km).

The clustering of households’ coordinates allows us to calculate the distance from the nearest cell tower at the community level only. To check whether this might affect our results, we perform community-level analysis by averaging women’s characteristics at the community level. The impact of distance to cell towers remains highly significant (*P*<.001). The magnitude of the impact also remains consistent, with a 0.8-percentage point decrease in the probability of knowing the testing facility (0.1 percentage points for probit). Underidentification and weak-instrument (Kleibergen-Paap Wald *F* statistic) tests for all IV regressions show that our instrument is relevant and reasonably strong.

**Table 2:** Impact of phone connectivity on knowledge of HIV testing facilities.

	Knowledge of HIV testing facility				
	Individual level			Community level	
	Probit	IV^a,b^ (2SLS^c^)	Probit		IV^d^ (2SLS)
Distance from the nearest cell tower (km) (cluster-robust SEs)	–0.002^e^ (<0.001)	–0.007^e^ (0.002)	–0.001^e^ (<0.001)		–0.008^e^ (0.002)
Individual-level characteristic	Yes	Yes	—^f^		—
Community-level characteristics	Yes	Yes	Yes		Yes
Country-year-fixed effects	Yes	Yes	Yes		Yes
Constant (cluster-robust SEs)	–0.547^e^ (0.058)	0.505^e^ (0.026)	0.304^e^ (0.021)		0.399^e^ (0.038)
Observations	351,613	351,488	18,437		18,431
*R* ^2^	0.307	0.183	0.682		0.402

^a^IV: instrumental variable.

^b^Underidentification (*P* value) test: <.001 and weak-instrument (Kleibergen-Paap rk Wald *F* statistic) test: 32.13.

^c^2SLS: 2-stage least squares.

^d^Underidentification (*P* value) test: <.001 and weak-instrument (Kleibergen-Paap rk Wald *F* statistic) test: 28.74.

^e^*P*<.001.

^f^Not available.

### From Knowledge to Action—Mobile Connectivity and HIV Testing

We have identified a significantly positive effect of proximity to cell towers on knowledge of HIV-testing facilities. It is important to also determine whether knowledge acquired through mobile phones ultimately translates into a behavioral change. We therefore investigate whether women living closer to cell towers not only acquire knowledge on where HIV testing can be conducted but also get tested.

Newly acquired knowledge affects individuals’ health behavior through what has been called the knowledge-attitude-behavior continuum [41]. Studies show that when agents act on the way information is processed by individuals, one could expect desired behavioral changes to ultimately happen [41]. Activities aimed at increasing knowledge about HIV transmission among pregnant women have been shown to change attitudes and behavior toward HIV testing [42,43]. At the same time, access to peer networks can influence individuals’ attitudes toward HIV testing. Studies in the past have found that peer-based interventions can increase HIV knowledge and adherence to AIDS treatment [44-46].

We examine whether better mobile connectivity has a direct effect on women’s probability of ever been tested for HIV. Our analysis is operationalized in a variation of equations 1 and 3, where having tested for HIV is now the dependent variable, and the explanatory variable is the distance from the nearest cell tower. Table 3 reports the results of our analysis. We find that a unit increase in distance from cell towers significantly reduces women’s probability of ever been tested for HIV by 0.5 percentage points in the individual-level model and by 0.4 percentage points in the community-level model. Again, we present in Figure S2 in Multimedia Appendix 1 a graphical illustration of our IV model’s predicted share of women tested by deciles of distance to the nearest cell tower. Similarly to the knowledge of testing facilities, the share of tested women is constant at around 70% in the top 4 deciles. At the start of the bottom 2 deciles, the share of tested women is already below 60%, and it reaches approximately 10% in the lowest decile.

**Table 3:** Impact of phone connectivity on HIV testing uptake.

	Ever tested for HIV			
	Individual level		Community level	
	Probit	IV^a,b^	Probit	IV^c^
Distance from the nearest cell tower (km) (cluster-robust SEs)	–0.005^d^ (0.001)	–0.005^e^ (0.002)	–0.001^d^ (<0.001)	–0.005^d^ (0.002)
Individual-level characteristic	Yes	Yes	—^f^	—
Community-level characteristics	Yes	Yes	Yes	Yes
Country-year-fixed effects	Yes	Yes	Yes	Yes
Constant (cluster-robust SEs)	–1.383^d^ (0.050)	0.147^d^ (0.025)	–0.036^g^ (0.019)	0.014 (0.029)
Observations	365,108	364,982	18,480	18,474
*R* ^2^	0.323	0.343	0.793	0.734

^a^IV: instrumental variable.

^b^Underidentification (*P* value) test: <.001 and weak-instrument (Kleibergen-Paap rk Wald *F* statistic) test: 34.21.

^c^Underidentification (*P* value) test: <.001 and weak-instrument (Kleibergen-Paap rk Wald *F* statistic) test: 29.08.

^d^*P*<.001.

^e^*P*=.02.

^f^Not available.

^g^*P*=.08.

### Robustness Checks

#### Reduced Number of Cell Towers

As a first robustness check, we examine whether the results remain significant when the distance from the nearest cell tower is calculated using only the cell towers inserted in the OpenCelliD dataset starting from the year in which each DHS was collected. Several countries are dropped from this analysis due to the complete absence of data on cell towers from the year of the survey onward, namely, Cameroon, Comoros, Gabon, Guinea, Mozambique, and Senegal. However, the figures presented in Table 4 show that results remain significant and consistent with previous findings.

**Table 4:** Impact of phone connectivity on HIV testing (restricted cell towers’ sample).

	Knowledge of HIV testing facility					Ever tested for HIV			
	Individual level		Community level		Individual level			Community level	
	OLS^a^	IV^b,c^	OLS	IV^d^	OLS		IV^e^	OLS	IV^f^
Distance from the nearest cell tower (km) (cluster-robust SEs)	–<0.001^g^ (<0.001)	–0.003^h^ (0.001)	–<0.001^h^ (<0.001)	–0.002^h^ (<0.001)	–<0.001^h^ (<0.001)		–0.002^i^ (0.001)	–<0.001^h^ (<0.001)	–0.001^j^ (<0.001)
Individual-level characteristic	Yes	Yes	—^k^	—	Yes		Yes	—	—
Community-level characteristics	Yes	Yes	Yes	Yes	Yes		Yes	Yes	Yes
Country-year-fixed effects	Yes	Yes	Yes	Yes	Yes		Yes	Yes	Yes
Constant (cluster-robust SEs)	–0.636^h^ (0.066)	0.475^h^ (0.025)	0.335^h^ (0.025)	0.369^h^ (0.041)	–1.568^h^ (0.059)		0.118^h^ (0.023)	–0.043^l^ (0.022)	–0.019 (0.032)
Observations	247,902	247,777	12,867	12,861	261,802		261,676	12,910	12,904
Pseudo-*R*^2^	0.315	0.0897	0.676	0.234	0.354		0.312	0.814	0.643

^a^OLS: ordinary least square.

^b^IV: instrumental variable.

^c^Underidentification (*P* value) test: <.001 and weak-instrument (Kleibergen-Paap rk Wald *F* statistic) test: 41.26.

^d^Underidentification (*P* value) test: <.001 and weak-instrument (Kleibergen-Paap rk Wald *F* statistic) test: 13.48.

^e^Underidentification (*P* value) test: <.001 and weak-instrument (Kleibergen-Paap rk Wald *F* statistic) test: 43.80.

^f^Underidentification (*P* value) test: <.001 and weak-instrument (Kleibergen-Paap rk Wald *F* statistic) test: 13.41.

^g^*P*=.84.

^h^*P*<.001.

^i^*P*=.004.

^j^*P*=.007.

^k^Not available.

^l^*P*=.74.

#### Impact on Other Health Care Seeking and Use

One question that can arise when looking at our results is whether the impact of mobile connectivity on knowledge of HIV-testing facilities and testing uptake is driven by a general impact of access to mobile phones on health seeking and use. For example, better connectivity may increase demand for health services from the population while at the same time reducing costs to access such services (eg, increased knowledge and better networking may provide individuals with more options for obtaining health care). To test whether this is the case, we repeat our analysis and replace our outcomes of interest with dummy variables for having health insurance and having delivered one’s last child in a safe structure (“safe structure” is defined here as either a public or a private health center). The results, presented in Table S3 in Multimedia Appendix 1, are not statistically significant in any of our preferred models (ie, 2SLS estimations). These results suggest that increased mobile connectivity does not have a general impact on health care seeking and use.

#### Alternative IV

As we have shown in our Statistical Methods section, terrain roughness and market competition are often associated with infrastructural development. However, there are additional characteristics that influence cell tower positioning in developed and developing countries alike. As an additional robustness check, we test the validity of lightning intensity and cost of mobile phone use as alternative instruments.

Frequent electrostatic discharges damage mobile phone infrastructures and negatively affect connectivity. Building cell towers in areas with high lightning intensity represents a risk for mobile phone provides since power surge protection for cell towers is costly and reduces profits from their investments. Being Africa the continent with the highest lightning intensity in the world, evidence has shown that a 1 SD increase in lightning intensity leads to a lower penetration rate of mobile phone technology of approximately 0.43 percentage points per year [47]. Mobile phone use depends on a variety of factors, of which cost is surely one of the most important [48]. Higher costs might be driven by low competition and, at the same time, act as an incentive for new companies to provide network access to a larger share of the population.

We interact the average intensity of lightning strikes in a 10-km radius around each DHS cluster with a country-level monthly cost of running a mobile phone as a percentage of per-capita income. Lightning strikes’ intensity data are publicly available at the Global Hydrology Resource Center. The satellite data are collected by the National Aeronautics and Space Administration’s Global Hydrology Resource Center. We included in our analysis yearly average lightning strikes for countries included in the analysis for 2010. Lightning strike incidence is proved to be stable through years, making the yearly average for 2010 a good proxy for the subsequent decade [47]. Information on the monthly cost of running mobile phones by country is retrieved from the 2015 International Telecommunication Union report on Measuring the Information Society. A slight modification to equation 2 produces

*D_i_* = π_0_ + π_1_*Flash intensity* × *Mobile cost_i_* + π_2_*Z_i_* + π_3_*Elec_i_* + π_4_*C* + ε*_i_*
**(4)**

Analysis conducted using the alternative IV produces results consistent with previous findings, which remain statistically significant (results presented in Table S4 in Multimedia Appendix 1).

### Mobile Phone Ownership Instead of Connectivity

We have focused our analysis on the impact of mobile network connectivity on women’s HIV and health knowledge and practices regardless of their ownership of a cellular phone. We did so because evidence has shown that mobile phone sharing is a common practice in sub-Saharan Africa [9]. It is the intention of the authors to argue for the potential of better phone connectivity regardless of increased ownership, and our results have supported our claims. However, it is logical to think that the easier the access to a mobile phone, the more relevant its impact on our outcomes of interest should be, and ownership of a mobile phone grants a much higher exposure to mobile technology than borrowing. DHS has recently started including questions on mobile phone ownership in its questionnaires as part of individuals’ and households’ assets. This provides us with a reduced sample of women who reported whether or not they own a cellular phone. Information on phone ownership is present for Angola (2015), Burundi (2016), Ethiopia (2016), Malawi (2015), Tanzania (2015), Uganda (2016), and Zimbabwe (2015).

To analyze the role of phone ownership, we make use of probit models as in equation 1, while we specify a new probit-2SLS model for the IV analysis (in this case, we use the same instrument from our main analysis, the interaction between land gradient and number of network providers). Given the binary nature of the phone ownership variable, a binary probit model for the first stage regression is more adequate. We present the results in Table 5. According to our IV estimation, phone ownership increases the chance of knowing a facility that conducts HIV tests by 8 percentage points, and it increases the chance of ever been tested for HIV by approximately 14 percentage points. Considering the percentage of people knowledgeable about HIV-testing place and ever tested for HIV who do not own a phone, our percentage points translate into a 9% higher chance of knowing a testing facility and a 22% higher chance of having tested for HIV for mobile phone owners.

**Table 5:** Phone ownership and HIV testing.

	Knowledge of HIV testing facility		Ever tested for HIV	
	Probit	IV^a^	Probit	IV
Mobile phone owner (cluster-robust SEs)	0.220^b^ (0.040)	0.078^b^ (0.016)	0.240^b^ (0.028)	0.142^b^ (0.025)
Individual-level characteristic	Yes	Yes	Yes	Yes
Community-level characteristics	Yes	Yes	Yes	Yes
Country-year-fixed effect	Yes	Yes	Yes	Yes
Constant (cluster-robust SEs)	–0.765^b^ (0.148)	0.708^b^ (0.012)	–1.654^b^ (0.101)	0.183^b^ (0.015)
Observations	76,241	76,241	81,048	81,048
Pseudo-*R*^2^	0.300	0.183	0.318	0.320

^a^IV: instrumental variable.

^b^*P*<.001.

### Mechanisms for Impact

Cell phone connectivity has been found to positively impact the probability of knowing HIV-testing facilities and of ever been tested for HIV. Through which mechanisms such impact is achieved is what we investigate in this section. To do so, we need to understand the barriers to HIV testing that mobile phone connectivity might help overcome. Studies have shown that stigma, both self and social, remains the most significant barrier to HIV testing in sub-Saharan Africa [49,50]. The provision of HIV counseling for patients at high risk has yielded good results among adolescents thanks to increased knowledge and awareness, although cultural change is hard to achieve [51]. Physical barriers to HIV testing are also limiting the effectiveness of awareness-raising programs. Distance from health facilities, inconvenient opening hours, and long waiting lines are among the reasons cited for low test uptake [51].

Mobile connectivity has been proven to be a powerful tool to spread information and increase social cohesion in the Global South [52]. Mobile phones also facilitate knowledge diffusion, which in turn reduces information asymmetry [7,53]. We argue that, while better mobile phone connectivity might do little to reduce physical barriers to HIV testing, it can increase women’s knowledge of HIV-related symptoms, help fight HIV misconceptions, and foster reproductive health knowledge.

To test our hypotheses, we perform an analysis on the role of distance from the nearest cell tower on several intermediate outcomes. We use knowledge of HIV and STIs and knowledge of modern contraceptive methods as dependent variables. HIV knowledge is measured, following standard DHS guidelines, as an index ranging from 0 to 1 measuring knowledge of HIV symptoms and preventive measures [54]. Knowledge of STIs and modern contraceptive methods are dummies assigning value 1 to knowledgeable women. The results of the analysis are presented in Table 6.

Distance from cell towers appears to have a significantly negative impact on HIV- and STI-related knowledge. In the same way, it also reduces the chance that a woman would be knowledgeable about modern contraceptive methods. These results seem to confirm our hypothesis that better cellular connectivity facilitates the acquisition of knowledge on reproductive health and sexually transmittable diseases. These findings are supported by previous evidence on the role played by mobile phones in knowledge-sharing and learning [15,55,56].

**Table 6:** Analysis of mechanisms of cell connectivity and HIV testing correlation.

	Knowledge of HIV			Knowledge of STIs^a^			Knowledge of contraceptive method	
	OLS^b^	IV^c,d^	Probit		IV^e^	Probit		IV^e^
Distance from the nearest cell tower (km) (cluster-robust SEs)	<–0.001^f^ (<0.001)	–0.004^g^ (0.001)	–0.003^g^ (0.001)		–0.002^g^ (0.001)	–0.002^g^ (0.001)		–0.004^g^ (0.001)
Individual-level characteristic	Yes	Yes	Yes		Yes	Yes		Yes
Community-level characteristics	Yes	Yes	Yes		Yes	Yes		Yes
Country-year-fixed effect	Yes	Yes	Yes		Yes	Yes		Yes
Constant (cluster-robust SEs)	0.577^g^ (0.012)	0.760^g^ (0.022)	–0.153^h^ (0.077)		0.741^g^ (0.016)	–0.660^g^ (0.069)		0.640^g^ (0.018)
Observations	340,062	339,965	359,431		368,812	369,000		368,874
Pseudo-*R*^2^	0.222	0.114	0.258		0.0505	0.345		0.0784

^a^STI: sexually transmittable infection.

^b^OLS: ordinary least square.

^c^IV: instrumental variable.

^d^Underidentification (*P* value) test: <.001 and weak-instrument (Kleibergen-Paap rk Wald *F* statistic) test: 31.52.

^e^Underidentification (*P* value) test: <.001 and weak-instrument (Kleibergen-Paap rk Wald *F* statistic) test: 34.01.

^f^*P*=.02.

^g^*P*<.001.

^h^*P*=.03.

To complete our analysis on mechanisms, we test whether results remain consistent when considering mobile phone ownership instead of mobile connectivity. Findings are reported in Table 7. Our results show that phone ownership significantly increases knowledge about STIs and modern contraceptive methods, while it seems to have no statistically significant impact on general knowledge of HIV.

Recent evidence has shown that mobile phone ownership might have an impact on contraceptive use and knowledge about family planning [57] and facilitate the spread of reproductive, maternal, newborn, and child health in vulnerable settings [58]. At the same time, some research has argued that better outcomes for phone owners are mostly driven by their economic status [59]. In our analysis, we control for additional factors that could influence women’s knowledge, such as media exposure, education, asset-based wealth, and place of residence, and the effect of mobile ownership remains statistically significant.

**Table 7:** Phone ownership and mechanisms.

	Knowledge of HIV		Knowledge of STIs^a^			Knowledge of contraceptive method	
	Probit	IV^b^	Probit	IV	Probit		IV
Mobile phone owner (cluster-robust SEs)	0.012^c^ (0.005)	0.006^d^ (0.013)	0.243^c^ (0.050)	0.053^c^ (0.016)	0.193^c^ (0.051)		0.089^c^ (0.015)
Individual-level characteristic	Yes	Yes	Yes	Yes	Yes		Yes
Community-level characteristics	Yes	Yes	Yes	Yes	Yes		Yes
Country-year-fixed effect	Yes	Yes	Yes	Yes	Yes		Yes
Constant (cluster-robust SEs)	0.651^c^ (0.019)	0.702^c^ (0.011)	–0.527^c^ (0.129)	0.722^c^ (0.014)	–0.297^e^ (0.137)		0.733^c^ (0.014)
Observations	66,344	66,344	81,048	81,048	81,048		81,048
Pseudo-*R*^2^	—^f^	—	0.325	—	0.478		—
*R* ^2^	0.296	0.239	—	0.174	—		0.236

^a^STI: sexually transmittable infection.

^b^IV: instrumental variable.

^c^*P*<.001.

^d^*P*=.64.

^e^*P*=.03.

^f^Not available.

## Discussion

### Principal Findings

This paper analyses the impact of cell phone connectivity on women’s HIV-testing knowledge and behavior. We used distance from the nearest cell tower as a proxy for phone connectivity strength and quality, and we estimated how increasing distance from said cell tower affects the probability of knowing where one can be tested for HIV and the probability of having taken such a test at least once in one’s life. Mobile phones enhance individuals’ access to information thanks primarily to knowledge-sharing through communication, although they can also put people in contact with institutional resources [15,56]. Due to phone-sharing practices in sub-Saharan Africa, we used cell phone connectivity as the primary indicator for analysis, with mobile phone ownership used for robustness check. We attempt to expand on the work that has been done on a smaller scale on the role played by mobile technology on HIV awareness-raising and testing [19,21]. We make use of a pooled cross-sectional dataset from 29 sub-Saharan African countries and different methods of analysis to produce robust inferences on the role played by phone connectivity.

Results show that mobile connectivity has a statistically significant and positive impact on the knowledge of HIV-testing facilities and having been tested for HIV, highlighting its role as an important determinant of these practices. Results are consistent in all models and at both individual and community levels. We test the hypothesis and find evidence that the impact of mobile phone connectivity on HIV-testing knowledge and behavior might be driven by improved knowledge of HIV, STIs, and contraceptive methods. Moreover, we find that the positive effects of mobile technology are even greater when we consider phone ownership as the explanatory variable.

### Comparison to Prior Work

Our results are in agreement with previous evidence that shows the role mobile technology can play in raising awareness about HIV and HIV testing [60]. The study also fits well into the new body of literature investigating mobile health and the role it could play in developing countries. Promising evidence shows how informal mobile health and access to mobile phones among young Africans can help bridge health care gaps in countries such as Ghana, Malawi, and South Africa [61,62]. Not only mobile phones but also all digital technology has been found to be beneficial for treating and preventing mental disorders in low-income and middle-income countries [63]. Our results support the idea that stronger mobile connectivity can foster HIV-testing practices in sub-Saharan Africa.

### Strength and Limitations

Our study is not exempt from limitations. First, although we are able to establish a link between mobile connectivity and knowledge of HIV-testing facilities and mobile connectivity and HIV-testing uptake, due to data limitations, we cannot dig deeper into how mobile connectivity shapes women’s attitudes toward testing, since no questions were asked to interviewed women about their opinion of HIV testing. Second, the OpenCelliD dataset is a relatively new dataset that might be lacking detail especially for remote areas. Missing information on the date in which cell towers are built also poses a limitation. We addressed both these issues by only including cell towers with the most reliable GPS coordinates and by performing robustness checks that aligned the building year of the cell towers with the year of the survey. When and if data will be available in the future, additional longitudinal analysis could be conducted to investigate the evolution of HIV-testing behavior through time for women with better mobile connectivity. A third limitation is represented by the clustering of individuals’ positions under 1 GPS coordinate representing the community. This clustering allows distance from the cell tower to be calculated only at the community level. We show, however, that our results remain significant even if individual figures are averaged at the cluster level, and community-level analysis is performed.

### Future Directions

Our study provides useful insights on the potential that new communication technologies represent to foster better reproductive health practices in sub-Saharan Africa and paves the way for future analysis on how mobile phones enhance knowledge-sharing around HIV/AIDS knowledge, behavior, and practices.

## Appendix

Multimedia Appendix 1 Additional robustness tests.

## Abbreviations

2SLS: 2-stage least squares
DCW: Digital Chart of the World
DHS: Demographic and Health Survey
GSM: Global System for Mobile Communication
IV: instrumental variable
STI: sexually transmittable infection

## Data Availability

All data used in this study are available on the Demographic and Health Survey repository, pending authorization, and the OpenCelliD repository.
