# Supplementary material for: Mobile Phones and HIV Testing: Multicountry Instrumental Variable Analysis From Sub-Saharan Africa
Source: J Med Internet Res. 2024 Sep 27;26:e48794. doi: 10.2196/48794 (PMC11470219; doi:10.2196/48794)
Supplement: Multimedia Appendix 1 [file jmir_v26i1e48794_app1.docx]

**Figure S1**. Differences between DCW and gROADS datasets.


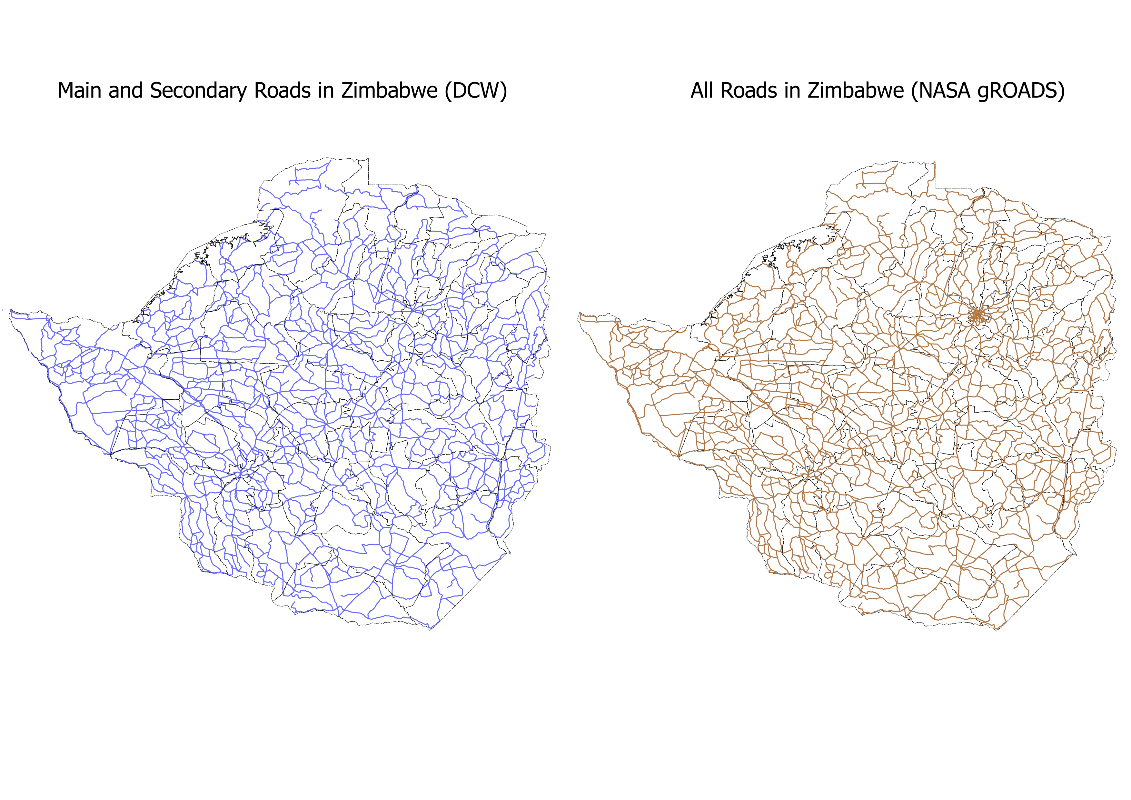


**Note**: Comparisons between the two road networks show minimum differences between the two datasets, which could be driven by the fact that in gROADS dataset the authors could not exclude trails from the list of roadways.

**Figure S2**. Predicted knowledge of testing facilities and testing attitude by deciles of distance to nearest cell tower.


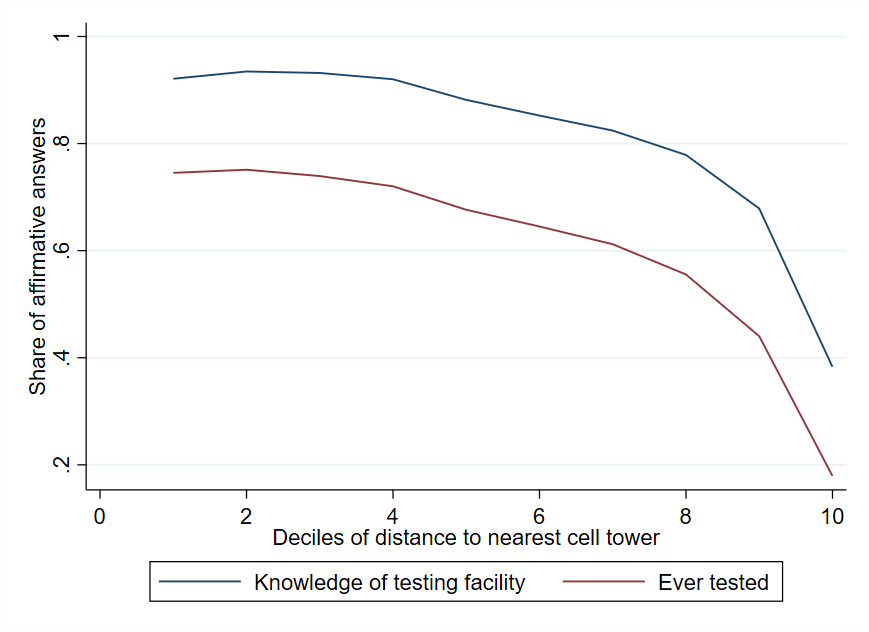


**Table S1**. First placebo test.

|  | **Knowledge of HIV testing facility** | | **Ever tested for HIV** | |
| --- | --- | --- | --- | --- |
|  | *Covered* | *Non-covered* | *Covered* | *Non-covered* |
|  | OLS | OLS | OLS | OLS |
| IV | 0.001*** | -<0.001 (P=0.90) | 0.001** | <-0.001 (P=0.55) |
|  | [0.001] | [0.001] | [<0.001] | [0.001] |
| Individual-level characteristic | Yes | Yes | Yes | Yes |
| Community-level characteristics | Yes | Yes | Yes | Yes |
| Country-year-fixed effects | Yes | Yes | Yes | Yes |
| Constant | 0.459*** | 0.225*** | 0.112*** | 0.013 |
|  | [0.015] | [0.070] | [0.014] | [0.043] |
|  |  |  |  |  |
| Observations | 322,902 | 28,586 | 332,153 | 32,829 |

**Note**: “Covered” and “Non-covered” refer to whether the area is covered or not by mobile network. Cluster-robust standard errors in brackets. *** p<.001.

**Table S2**. Second placebo test.

|  | **Exposed to any type of media** | | **Distance to nearest road (km)** | | **Population density** | |
| --- | --- | --- | --- | --- | --- | --- |
|  | OLS | IV | OLS | IV | OLS | IV |
| IV | <0.001 (P=0.34) |  | 0.006 (P=0.54) |  | -2.450 (P=0.37) |  |
|  | [<0.001] |  | [0.010] |  | [2.752] |  |
| Distance from nearest cell (km) |  | -0.004 (P=0.38) |  | -0.121 (P=0.57) |  | 48.130 (P=0.42) |
|  |  | [0.004] |  | [0.213] |  | [59.449] |
| Individual-level characteristic | Yes | Yes | Yes | Yes | Yes | Yes |
| Community-level characteristics | Yes | Yes | Yes | Yes | Yes | Yes |
| Country-year-fixed effects | Yes | Yes | Yes | Yes | Yes | Yes |
| Constant | 0.464*** | 0.545*** | 5.966*** | 8.691* | 1,170.783** | 86.896 |
|  | [0.011] | [0.092] | [0.358] | [4.724] | [495.772] | [1,381.918] |
|  |  |  |  |  |  |  |
| Observations | 404,718 | 404,718 | 404,745 | 404,745 | 404,745 | 404,745 |

**Note**: Cluster-robust standard errors in brackets. *** p<.01, ** p<.05, * p<.1.

**Table S3**. Alternative outcomes for healthcare demand.

|  | **Has health insurance** | | **Safe delivery of last pregnancy** | |
| --- | --- | --- | --- | --- |
|  | Probit | IV | Probit | IV |
| Distance from nearest cell (km) | -0.003*** | <-0.001 (P=0.82) | -0.004*** | <0.001 (P=0.78) |
|  | [0.001] | [0.001] | [0.001] | [0.001] |
| Individual-level characteristic | Yes | Yes | Yes | Yes |
| Community-level characteristics | Yes | Yes | Yes | Yes |
| Country-year-fixed effect | Yes | Yes | Yes | Yes |
| Constant | -2.650*** | -0.019** | -0.931*** | 0.216*** |
|  | [0.077] | [0.009] | [0.050] | [0.019] |
| Observations | 330,496 | 330,370 | 232,334 | 232,269 |
| (pseudo) R-squared | 0.338 | 0.269 | 0.334 | 0.378 |
| Underidentification (p-value) |  | <0.001 |  | <0.001 |
| Kleibergen-Paap rk Wald F statistic |  | 34.39 |  | 34.30 |

**Note**: Cluster-robust standard errors in brackets. *** p<.001. Underidentification and weak-instrument (Kleibergen-Paap rk Wald F statistic) tests reported at bottom of the table for IV regressions.

**Table S4**. Alternative instrumental variable.

|  | **Knowledge of HIV testing facility** | **Ever tested for HIV** |
| --- | --- | --- |
|  | IV | IV |
| Distance from nearest cell (km) | -0.002 (P=0.08) | -0.002*** |
|  | [0.001] | [0.001] |
| Individual-level characteristic | Yes | Yes |
| Community-level characteristics | Yes | Yes |
| Country-year-fixed effects | Yes | Yes |
| Constant | 0.457*** | 0.125*** |
|  | [0.017] | [0.016] |
| Observations | 351,613 | 365,108 |
| R-squared | 0.273 | 0.365 |
| Underidentification (p-value) | <0.001 | <0.001 |
| Kleibergen-Paap rk Wald F statistic | 32.44 | 29.36 |

**Note**: Cluster-robust standard errors in brackets. *** p<.001. Underidentification and weak-instrument (Kleibergen-Paap rk Wald F statistic) tests reported at bottom of the table for IV regressions.
